# Hello, is it me you’re Stopping for? The Effect of external Human Machine Interface Familiarity on Pedestrians’ Crossing Behaviour in an Ambiguous Situation

**DOI:** 10.1177/00187208241272070

**Published:** 2024-08-23

**Authors:** Yee Mun Lee, Vladislav Sidorov, Ruth Madigan, Jorge Garcia de Pedro, Gustav Markkula, Natasha Merat

**Affiliations:** 1 4468 University of Leeds, UK

**Keywords:** automated vehicles, external human–machine interface, interaction, pedestrians, crossing, malfunction, deceleration, intention

## Abstract

**Objective:**

We investigated how different deceleration intentions (i.e. an automated vehicle either decelerated for leading traffic or yielded for pedestrians) and a novel (Slow Pulsing Light Band – SPLB) or familiar (Flashing Headlights – FH) external Human Machine Interface (eHMI) informed pedestrians’ crossing behaviour.

**Background:**

The introduction of SAE Level 4 Automated Vehicles (AVs) has recently fuelled interest in new forms of explicit communication via eHMIs, to improve the interaction between AVs and surrounding road users. Before implementing these eHMIs, it is necessary to understand how pedestrians use them to inform their crossing decisions.

**Method:**

Thirty participants took part in the study using a Head-Mounted Display. The independent variables were deceleration intentions and eHMI design. The percentage of crossings, collision frequency and crossing initiation time across trials were measured.

**Results:**

Pedestrians were able to identify the intentions of a decelerating vehicle, using implicit cues, with more crossings made when the approaching vehicles were yielding to them. They were also more likely to cross when a familiar eHMI was presented, compared to a novel one or no eHMI, regardless of the vehicle’s intention. Finally, participants learned to take a more cautious approach as trials progressed, and not to base their decisions solely on the eHMI.

**Conclusion:**

A familiar eHMI led to early crossings regardless of the vehicle’s intention but also led to a higher collision frequency than a novel eHMI.

**Application:**

To achieve safe and acceptable interactions with AVs, it is important to provide eHMIs that are congruent with road users’ expectations.

## Introduction

According to the WHO (2018), among vulnerable road users, pedestrians represent a quarter of all global road traffic deaths, with more than 1/3 of fatalities happening in urban areas (European Commission, 2018). Recent rapid technological developments in Automated Vehicles (AV) are expected to improve road users’ safety, and minimise fatalities. This is because AVs are expected to avoid the mistakes caused by drivers, such as those brought about by impairments due to fatigue or distraction (Clamann et al., 2017). The introduction of Level 4 automation (SAE, 2018) removes the possibility of pedestrian-driver communication, and a new type of interaction is likely to be formed between pedestrians and AVs. However, these new forms of interaction can also result in new challenges for communication in a mixed traffic environment with AVs, caused by issues such as pedestrians’ inability to understand the meaning and intentions of external Human–Machine Interfaces (eHMI) (Lee et al., 2019, 2022), or unsafe behaviour due to misunderstandings or excessive trust of the message provided to pedestrians (Kaleefathulah et al., 2020).

Studies suggest that while interacting with a conventional vehicle/driver, pedestrians depend on implicit communication methods for their crossing decisions, such as the speed of an approaching vehicle, its time-to-arrival, and stopping distance (Ackermann et al., 2019a; Dey & Terken, 2017; Domeyer et al., 2022; Lee et al., 2021; Petzoldt et al., 2018; Sucha et al., 2017; Uttley et al., 2020; Várhelyi, 1998; Wang et al., 2014). Explicit communication, such as hand and head gestures, honking of the horn, and flashing headlights, have also been shown to play a role in these interactions, although to a much lesser extent (Mahadevan et al., 2018; Rasouli et al., 2017; Šucha et al., 2017). Given that driver-based communications will not be possible for higher levels of AVs, a number of OEMs have recently considered the inclusion of new eHMI concepts to aid explicit communication between future AVs and other road users (e.g. Daimler, 2015; Daimler, 2017; Drive.ai, 2018; Jaguar Land Rover, 2018; Nissan Motor Corporation, 2015; Volvo Car, 2018). There has also been a recent rise in research interest in this area (Bazilinskyy et al., 2019; Dey et al., 2020; Rasouli & Tsotsos, 2019; Schieben et al., 2019). For example, eHMIs have been shown to increase user acceptance and trust, their willingness to interact with the AVs, and improve understanding of AV’s intentions (e.g. Böckle et al., 2017; Deb et al., 2018; Kooijman et al., 2019; Monzel et al., 2021; Petzoldt et al., 2018). Automated vehicles equipped with eHMIs are also shown to help pedestrians make quicker crossing decisions, thus saving time and improving overall traffic flow (e.g. Chang et al., 2017; Holländer, Wintersberger, & Butz, 2019; Lee et al., 2022; Pekkanen et al., 2021).

Research has shown that the use of an eHMI can reduce pedestrians’ Crossing Initiation Time (CIT), especially if they were familiar with the eHMI design (i.e. a flashing headlight) and if the eHMI is presented at lower vehicle approach speeds and time gaps (Lee et al., 2022). eHMIs also appear to be most effective when their messages are congruent with the implicit cues provided by the vehicle (Dey et al., 2020; Lee & Sheppard, 2016). On the other hand, Kaleefathullah et al. (2020) found that, after a series of exposures to approaching vehicles with an eHMI, pedestrians started to over-rely on this explicit form of communication, with about 30% of them colliding with the AV if the implicit cues were incongruent with the eHMI (i.e. eHMI was presented to indicate yielding, but the AV was not in fact yielding). Clearly, the consequences of such an over-reliance could be severe. Understanding how pedestrians use implicit and explicit information from an approaching vehicle for their crossing decisions, especially if these are incongruent, is an under-researched area.

The aim of the current study, therefore, was to extend our knowledge in this area, by focussing on a common traffic scenario which has not been studied to date. This involves an approaching vehicle that could be decelerating for many reasons, such as due to traffic buildup in its future path, or because it is yielding to other road users. In these circumstances, pedestrians who are interacting with a decelerating vehicle cannot assume that the vehicle is stopping or yielding for them. Therefore, our first research question was whether participants would be able to determine if an approaching vehicle was yielding for them, or decelerating because of a buildup of traffic further ahead.

Our second research question was whether, and how, this crossing is aided by the presence of novel (i.e. Slow Pulsing Light Band) versus a familiar (i.e. Flashing Headlights) eHMI (see also Lee et al., 2022). Information processing of eHMIs can be understood from both bottom-up (i.e. perception based on senses) and top-down (i.e. perception based on our knowledge and experience) processing perspectives (Lee et al., 2017). In this case, bottom-up processing refers to the saliency or visibility of the eHMI. Some research has suggested that crossing decisions are faster if an eHMI is easily perceivable from a distance, or if it can be preattentively processed (Holländer et al., 2019; Treisman & Souther, 1985). Lee et al. (2022) tested the distance at which the Slow Pulsing Light Band (SPLB) and Flashing Headlights (FH) eHMIs could be perceived. Their research found that FH was perceived at a further distance than SPLB. However, the lack of surge in crossing decisions at the moment SPLB could be perceived, led the authors to conclude that visibility is not the only reason for earlier crossings. This suggests that top-down processing of the eHMI, due to familiarity with, and comprehension of, the eHMI, also contributes to crossing decisions. Although FH is not a legally acceptable form of communicating yielding intent in the UK (Rule 110, 111, DFT, 2019, August 20), it is a commonly used and understood message for this behaviour (Driving Test Tips, 2021; Honest, 2004). We hypothesised that, due to limited human capabilities in identifying the deceleration profile of an approaching vehicle (e.g. Cavallo & Laurent, 1988; DeLucia, 2008; Hoffmann & Mortimer, 1994; Scialfa et al., 1991; Smeets et al., 1996), pedestrians would be very sensitive to the messages conveyed by an eHMI. They would be more likely to base their crossing decisions on the eHMI message, which is highly salient and consistent over time, than kinematic information, which demands focused attention over time. We anticipated that this effect would be stronger in response to the more familiar eHMI (FH).

Finally, the third aim of the study was to establish whether there are learning effects over time, regarding how much pedestrians rely on the information conveyed through an eHMI. If the eHMI of AVs is inconsistent with the kinematic information provided by the AV, one would expect human road users to adapt and become less likely to make a crossing decision based on the eHMI over time, but this has not been investigated in previous studies.

## Method

### Participants

This research was supported by project ‘interACT’ funded by the European Union’s Horizon 2020. Ethical approval was obtained from the University of Leeds Research Ethics Committee (Ref: LTTRAN-097). Participants were recruited by posting notices in the University’s students’ union building and via social media. Thirty participants (15 males, 15 females) with a mean age of 25.3 (Range = 20–36 years old; *SD* = 4.04) took part in the experiment. All participants had lived in the United Kingdom for at least ten months (Range = 10–288 months; *M* = 93.9, *SD* = 116.6). The study lasted 1 hour, and participants were paid £10 for their participation.

### Apparatus

**Figure 1.**
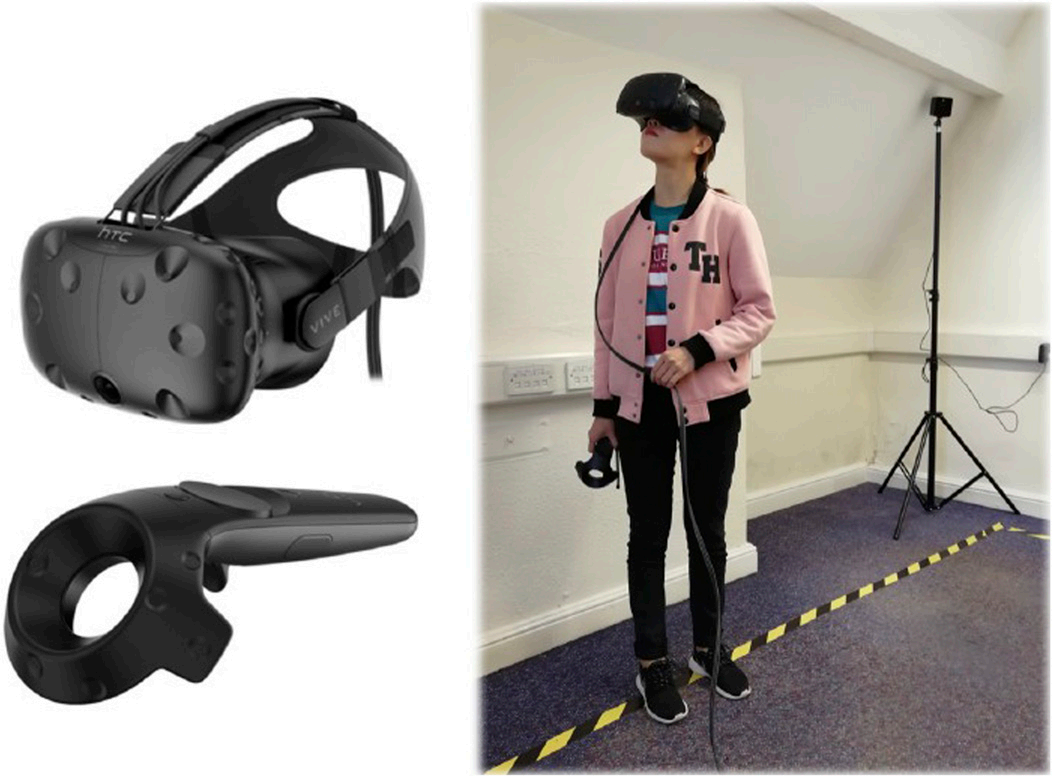
HTC VIVE head-mounted display (HMD) used in this study.

An HTC VIVE head-mounted display (HMD), incorporating a handheld wireless push-button controller was used to display the virtual environment for this study, which was created with the Unity cross-platform game engine (unity.com) (see Figure 1).

### Scenario

**Figure 2.**
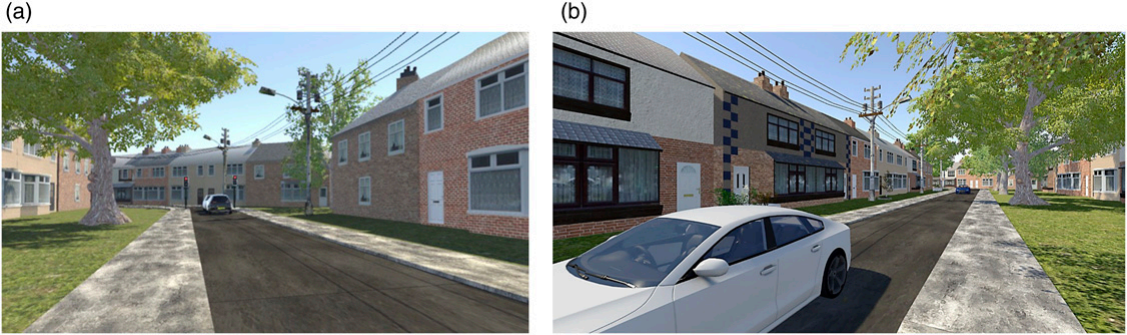
(a) Participants’ left view: Vehicles queuing and waiting at the traffic signal. (b) Participants’ right view: The two approaching vehicles.

A daytime urban environment, which included a 4.2 m wide single-lane road, was created, with houses located on both sides of the road (see Figure 2). A row of trees was created on one side of the road to indicate the pedestrians’ starting position before each crossing. Two bollards were positioned at each side of the road to guide participants' crossing paths.

**Figure 3.**
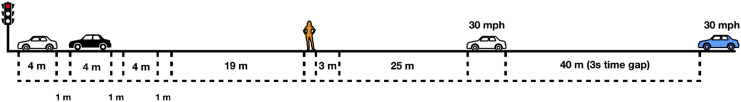
An illustration of the traffic scene seen by the pedestrians as they began each trial. The approaching vehicles to the right and the stationary vehicles waiting at the red traffic light to the left.

At the beginning of each trial, there were always two vehicles (one white and one black) waiting at the red traffic light, which was 34 m away, to the left of the pedestrian (Figure 2(a), and Figure 3). The intention here was to create ambiguity about whether the approaching vehicles (from the right) would yield for the pedestrian, or not, while decelerating to stop for the red traffic light. At the start of each trial, two vehicles (a white car followed by a blue car) approached from the right (Figure 2(b)). The first approaching vehicle appeared at 25 m away from pedestrians at the beginning of the trial. Three seconds later the second approaching vehicle appeared (positioned 40 m behind the white vehicle). Both vehicles travelled at a constant initial speed of 30 mph (13.4 m/s) (see Figure 3), creating the 3 s time gap between them. This two-vehicle method was used to maintain the same time gap for each crossing trial (see also Lee et al., 2022; Lobjois & Cavallo, 2007).

The first approaching vehicle always drove past the pedestrian to stop at the traffic light. When the rear of the first approaching vehicle passed the pedestrian, it began decelerating at a rate of 3.6 m/s^2^ to stop at the traffic lights, becoming the third vehicle joining the queue. At the same time, the second approaching vehicle also began decelerating, either to yield for the pedestrian (20% trials) or to decelerate to join the queue (80% trials). Thus, the second approaching vehicle started its deceleration at the same time in all trials, but displayed two different deceleration behaviours. A constant deceleration pattern was deployed, and the deceleration rates for ‘yielding for the pedestrian’, and ‘decelerating for the traffic lights’ were 2.43 m/s^2^ and 1.52 m/s^2^, respectively. In the trials where the approaching vehicle yielded for pedestrians, it stopped 3 m before arriving at the crossing path. This stopping distance was based on the UK Department for Transport’s Rule 126 (DFT, 2019, August 20). Participants were asked to cross between the two approaching vehicles, if they felt comfortable doing so.

### eHMI Designs

**Figure 4.**
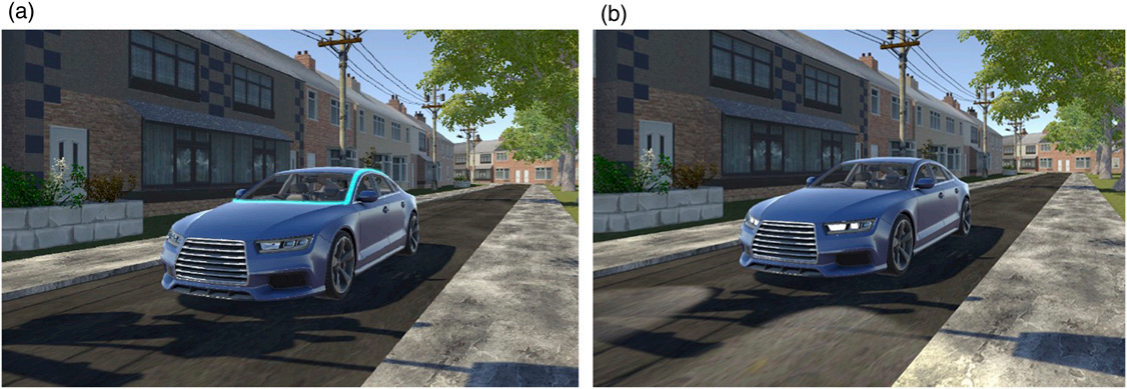
eHMI designs. (a) Slow Pulsing Light Band; (b) Flashing Headlights: Note the illumination on the road.

Two eHMI designs, Flashing Headlights (FH) and Slow Pulsing Light Band (SPLB), were used in this study, and the effect of these eHMIs on crossing decisions was tested. Both of these designs were associated with the message ‘I am giving way' in a paired forced choice and 5-point rating task study using an HMD (Lee et al., 2019). According to Lee et al. (2019), FH was ranked the highest of ten different eHMI designs for communicating the message: ‘I am giving way'. This study also found similar ranking of SPLB (M = 3.93) and FH (M = 4.18) for conveying the ‘I am giving way’ message. There was also no significant difference in Perceived Safety ratings while crossing in front of vehicles that presented these two eHMI designs. The SPLB, which was developed as part of the European project interACT (Weber et al., 2019), was a cyan light band presented at a pulsing rate of 0.4 Hz and placed around the front windscreen of the vehicle, as shown in Figure 4(a). The Flashing Headlight (Figure 4(b)) was implemented by using a combination of texture and Unity spotlights, turning on and off over a 300 ms period. Both eHMIs were always displayed at the onset of deceleration, with FH flashing three times on approach to the pedestrian’s position, and SPLB continuously pulsing slowly until the pedestrians passed the vehicle. No information was given about the meaning of the eHMI, which allowed us to study the learning effects for each eHMI.

### Experimental Design

A within-participant design was used, with two independent variables: (i) the deceleration behaviour of the second approaching vehicle (‘decelerating for the traffic lights’ or ‘yielding for the pedestrian’) and (ii) eHMI condition (No eHMI, SPLB, or FH, Figure 4). Each eHMI condition was presented in a block of 30 crossing trials, and a Latin square counterbalancing technique was used. For six of these trials (20%), the approaching vehicle was ‘decelerating for the traffic lights’, and for the remaining 24 trials (80%) it was ‘yielding for the pedestrian’ (see Table 1). This 80–20 split was used to create a situation where the approaching vehicle was yielding to the pedestrians most of the time, and the eHMI was reliable in communicating its intention for the majority of the time. Within each block, the six trials where the vehicle decelerated for the traffic lights were presented at the 5^th^, 12^th^, 16^th^, 22^nd^, 27^th^, and 30^th^ positions in the block. This order was the same for each block, allowing us to investigate learning effects across the experiment.

**Table 1.** Experimental Design, Conditions, and Number of Trials.

eHMI Condition	Decelerating Behaviour	Number of Trials (%)	Trial Number
No eHMI	Decelerating for the traffic lights	6 (20%)	5th, 12th, 16th, 22nd, 27th, 30th
	Yielding for the pedestrian	24 (80%)	All others
Slow pulsing light band (SPLB)	Decelerating for the traffic lights	6 (20%)	5th, 12th, 16th, 22nd, 27th, 30th
	Yielding for the pedestrian	24 (80%)	All others
Flashing headlights (FH)	Decelerating for the traffic lights	6 (20%)	5th, 12th, 16th, 22nd, 27th, 30th
	Yielding for the pedestrian	24 (80%)	All others

### Procedure

Upon arrival, participants gave their informed consent, and were given the opportunity to ask any questions. Written instructions were provided to the participants, accompanied by figures, as well as verbal explanations, as follows: *‘At the beginning of each trial, you will be standing at the pavement. On the left, you will see two cars standing in a queue at the traffic light. On the right, you will see two cars approaching. Your task is to cross naturally (or not to cross) between the two approaching cars (see figures below). After crossing the road, you will have to walk back to the starting position, triggering the next trial, again by pressing the controller. Please note that the researcher would like you to cross naturally as soon as you feel comfortable’.*

A practice block (with No eHMI) was administered to make sure participants understood the instructions of the task, providing them with the opportunity to familiarise themselves with the HMD and virtual environment. The practice block ended as soon as they understood and had familiarised themselves with the experiment, which usually took around three trials. If participants did cross the road, they were asked to cross back to their initial position after the car had passed, and to press the button to trigger the next trial. If participants did not cross the road, they were to press the button to trigger the next trial from their current position. Each experimental block took approximately 15 minutes to complete, after which, participants took a short break and were asked to complete the Misery scale, which measured motion sickness or unease during the experiment (Bos et al., 2010). A score of four or higher would suggest that the participant should not continue the study, but this did not occur.

## Results and Discussion

One participant was excluded from the data analysis because they were hit by the AV in 94% (*N* = 17 out of 18) of the ‘decelerating for the traffic lights’ trials. In total, 2610 trials of data were included in the analysis (29 participants × 90 trials). To understand crossing behaviour in response to the two eHMIs, and for the two different decelerating conditions, we calculated the percentage of crossings as a function of vehicle distance, the collision frequency when the vehicle was ‘decelerating for the traffic lights’ conditions, and whether there was any learning effect throughout the study, by investigating the changes in Crossing Initiation Time (CIT) across trials.

### Percentage of Crossings

Pedestrians crossed 100% of the time when the AV was yielding for them, which is not surprising, because the AV always stopped 3 m ahead of the pedestrian in those trials. When the AV was ‘decelerating for the traffic lights’, each of the three eHMI conditions led to a similar crossing rate, at 57%, 54%, and 57%, for the No eHMI, SPLB, and FH, respectively.

**Figure 5.**
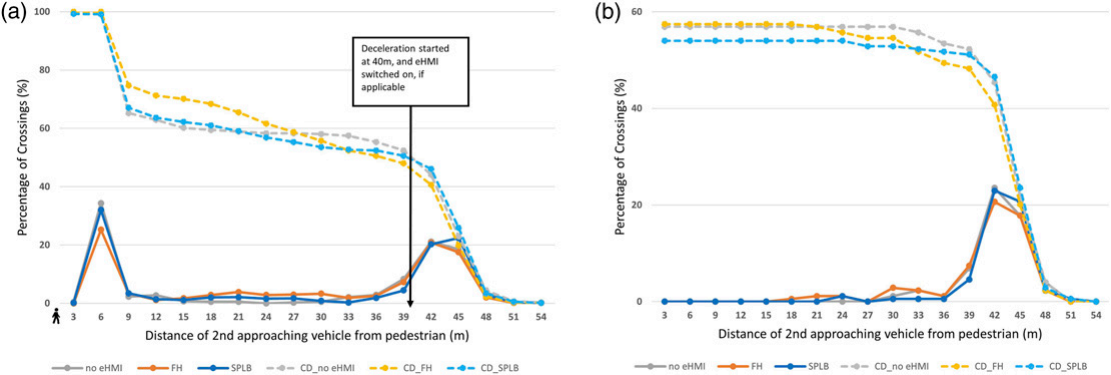
Percentage of crossings as a function of vehicle distance to the pedestrian, where dotted lines represent the cumulative distribution of the percentage of crossings. (a) ‘Yielding for the pedestrian’ trials. (b) ‘Decelerating for the traffic lights’ trials. For reference, TTAs for (a) at 3 m, 15 m, 27 m, and 39 m were 0 s, 1.45 s, 2.15 s, and 2.95 s respectively; and (b) 0.37 s, 1.47 s, 2.28 s, and 2.87 s, respectively.

Figure 5(a) and (b) show the percentage of crossings as a function of the distance of the vehicle to the pedestrian for both ‘yielding for the pedestrian’ and ‘decelerating for the traffic light’ trials. For ease of understanding, the crossings are plotted for each 3 m bin (the distance at which the yielding vehicle stopped), starting from 54 m away. The crossing gap opened when the first vehicle passed the pedestrians, at which point the second vehicle was 40 m away. Therefore, the first peak of crossings (rightmost peak) seen in both Figure 5(a), and (b), is associated with crossings that were initiated as soon as, or just before, the gap opened. In both decelerating conditions, for between 41% and 47% of the trials (cumulative percentage), pedestrians crossed when the vehicle was more than 39 m away, regardless of eHMI conditions.

As shown in Figure 5(a), in the ‘yielding for the pedestrian’ condition, a bimodal crossing distribution was observed, where the second crossing peak arose when the vehicle was nearly or completely stopped (between 3–6 m) (No eHMI: 35%; SPLB: 32%; FH: 25%). This bimodal pattern is a common phenomenon observed by a number of previous studies, whereby pedestrians mostly cross when the vehicle is very far away, or else they wait until the vehicle has come to a near or complete stop (see also Giles et al., 2019; Lee et al., 2019; Lee et al., 2022; Markkula et al., 2020; Schneemann & Gohl., 2016).

However, to investigate if participants were able to establish whether the approaching vehicle was yielding to them or ‘decelerating for the traffic lights’, and if the decision was influenced by the two eHMIs, we looked at any crossings made between the two peaks of road crossing, since this was where there was ambiguity about the AV’s decelerating behaviour. Results showed that the percentage of crossings that occurred when the approaching vehicle was between 6 m and 39 m away was 11% (No eHMI), 7% (SPLB), and 17% (FH), for the ‘decelerating for traffic lights’ condition, and 21% (No eHMI), 21% (SPLB), and 34% (FH), for the ‘yielding for the pedestrian’ condition.

**Figure 6.**
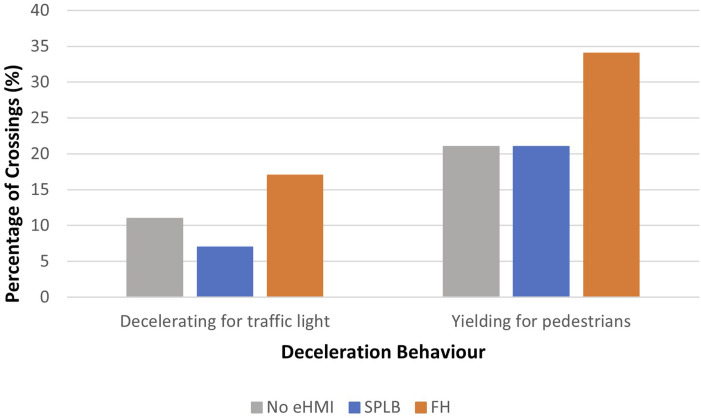
Percentage of crossings between the two peaks, for each eHMI and deceleration behaviour.

A Generalised Estimating Equation analysis (GEE) (Hong & Ottoboni, 2017; Liang & Zeger, 1986) was conducted to investigate the effect of decelerating intention and eHMIs on crossings that occurred between the two peaks (see Figure 6). The GEE accounts for the repeated measures design of the experiment. The working correlation matrix was specified as exchangeable in SPSS, and participant IDs were specified as subject variables in the model. Decelerating intention and eHMIs were indicated as the independent variables, and crossings between the two peaks (binary) were indicated as the dependent variable. The distribution and link function was specified as Binary Logistic. A set of post-hoc pairwise comparisons with Bonferroni corrections were conducted, to compare crossings made between eHMIs (three levels). The confidence interval was set to 0.05 for all analyses reported in this study.

The GEE analysis indicated a main effect of decelerating intention, x^2^ (1) = 11.27, *p* = .001. More than double the crossings were made when the vehicle was ‘yielding for the pedestrian’, compared to when the vehicle was ‘decelerating for the traffic lights’. This suggests that even before the vehicle had come to a near-complete stop, pedestrians were able to discriminate whether the approaching vehicle was decelerating for them, or for the traffic lights. However, the percentage of crossings between the two peaks in the ‘yielding for the pedestrian’ condition was still generally low compared to crossings made when the approaching vehicle had stopped, or nearly stopped.

There was also a main effect of eHMI, x^2^ (1) = 13.77, *p* = .001. Pairwise comparisons showed that more crossings were made between the two peaks in the FH condition, when compared to the SPLB condition (*p* = .002). More crossings were also made in the FH condition, compared to the No eHMI condition (*p* = .024); and no difference was found between the SPLB and the No eHMI condition (*p* = .457). No interactions were found.

We also found that the percentage of crossings between the two peaks was the highest in response to the FH, for both decelerating conditions, with little difference seen between the SPLB and No eHMI condition, supporting the hypothesis of our second research question. Based on our previous experiments, which found no link between the perception time of a novel eHMI and the resulting crossing behaviour (Lee et al., 2022), this result suggests that the familiarity of the eHMI is important for encouraging crossings. Lee et al. (2022) also demonstrated that the use of an FH eHMI led to earlier crossings, when compared to SPLB. However, as shown by Kaleefatullah et al. (2020), over-reliance on an eHMI may be dangerous, if this means pedestrians will pay less attention to a vehicle’s kinematic behaviour when making their crossing decisions. To consider this further, the safety implications of pedestrians’ crossing decisions and crash data were investigated for the three eHMI conditions, during the ‘deceleration for traffic light’ trials.

### Collision Frequency: ‘Decelerating for the Traffic Lights’ Condition

**Figure 7.**
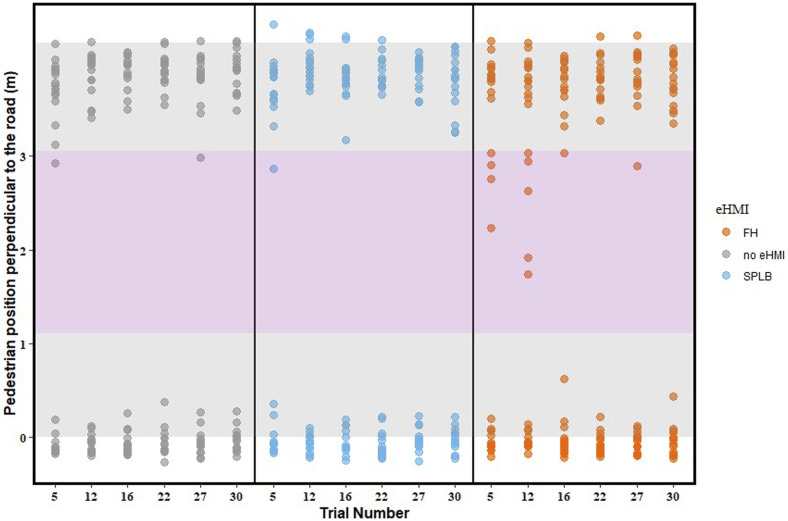
‘Decelerating for the traffic lights’ condition: Pedestrians’ position perpendicular to the road, at the moment when the front of the approaching vehicle was located at the crossing path. The grey area illustrates the road surface, while the pink area represents the vehicle’s width. The markers are transparent so that overlap can be distinguished.

To investigate pedestrian crossing safety in the ‘decelerating for the traffic lights’ trials, we calculated all pedestrians’ positions at the moment the front of the second vehicle arrived in their crossing path, by plotting their location for the relevant trials, that is, the 5^th^, 12^th^, 16^th^, 22^nd^, 27^th^, and 30^th^ trial (*x*-axis, see Figure 7). The *y*-axis depicts the road, and the dots closer to the *x*-axis (lower y) represent participants who had not yet crossed the road, whereas the dots located at the top of the figure represent pedestrians who had finished crossing the road (starts at 0 on the *y*-axis). The grey area depicts the road surface, and the pink area depicts the width of the vehicle.

The figure shows that there were 14 collisions in the whole experiment, as depicted by the 14 dots in the pink area when the vehicle overlapped with the pedestrians’ crossing path. Table 2 shows more details of the 14 collisions, 11 of which were in the FH condition, mainly in Blocks 1 and 2. A similar GEE analysis to the one described for percentage of crossings was conducted, with eHMI and collision outcome as independent and dependent variables, respectively. A set of post-hoc pairwise comparisons with Bonferroni corrections were conducted to compare collisions between eHMIs (three levels). Results revealed a significant main effect of eHMI, x^2^ (2) = 14.27, *p* = .001. Pairwise comparisons showed significantly more collisions in the FH condition, than for SPLB (*p* = .014), and No eHMI condition (*p* = .017), in line with our hypothesis for the second research question. This is because pedestrians are more inclined to make crossing decisions based on a more familiar eHMI (Lee et al., 2022). Block number was also included as a predictor in this analysis, to test for possible learning effects, but with this more advanced model, the GEE fitting procedure did not converge, so we are unable to draw any conclusions. However, it may be noted in Table 2 that 13 of the 14 collisions occurred in the first two blocks, and it can be seen in Figure 6 that 9 of the 11 collisions in the FH condition happened during the first and second trials within a block, suggesting that participants may have been more prone to collide early in the experiment, and early within blocks.

**Table 2.** Summary of the 14 Collisions.

No. of Collisions	Block 1	Block 2	Block 3	Total
No eHMI	1	1	0	2
FH.	4	7	0	11
SPLB	0	0	1	1

### Learning Effect: Change in CIT Across Trials

As noted above, the collision data suggested a possible learning effect. However, since collisions were rare, clear conclusions were not possible. Therefore, to further investigate our third research question on whether pedestrians learned to reduce their reliance on the information conveyed through an eHMI over time, we investigated the effect of each eHMI and trial number on the average Crossing Initiation Time (CIT), in both the ‘decelerating for the traffic lights’ and the ‘yielding for the pedestrian’ conditions, using GEE analysis. CIT was measured as the time taken for participants to start crossing the road after the rear end of the first vehicle had passed the crossing point, indicating how quickly a crossing decision was made (Lee et al., 2022; Lobjois & Cavallo, 2007, 2009).

Two GEEs were used to investigate the effect of eHMIs and blocks on CIT, one for each deceleration behaviour. Similar to previous GEE analysis, but with a linear link function used, eHMIs and block were indicated as the independent variables, and CIT was indicated as the dependent variable. A set of post-hoc pairwise comparisons with Bonferroni corrections were conducted to compare CIT between eHMIs (three levels).

**Figure 8.**
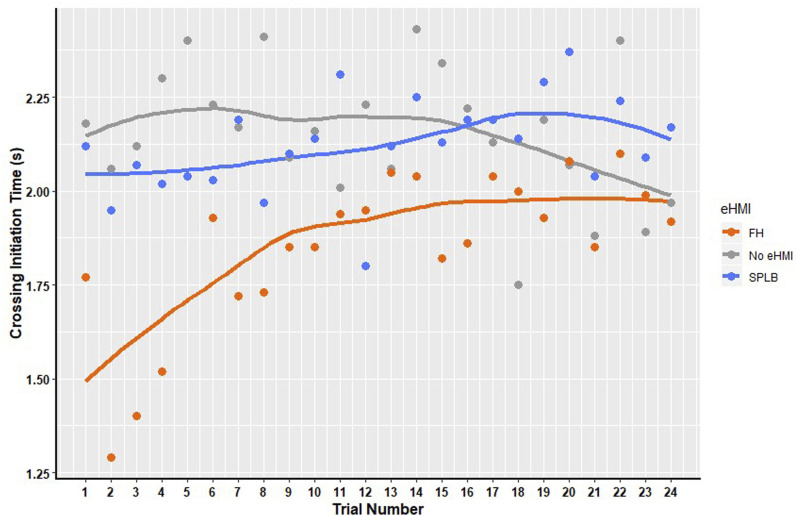
Mean of Crossing Initiation Time (s) across trials for FH, No eHMI and SPLB for ‘yielding for the pedestrian’ condition.

For the ‘yielding for the pedestrian’ condition (see Figure 8), there was no significant main effect of eHMI, x^2^ (2) = 5.47, *p* = .065, but there was a significant main effect of trial number x^2^ (23) = 77.72, *p* < .001 and an interaction between eHMI and trial number x^2^ (2) = 3.526 E+12, *p* < .001. To investigate whether CIT changed significantly across trials for each of the eHMI conditions, three GEE analyses were conducted, with all showing a significant change in CIT across trials (No eHMI: x^2^ (23) = 101.39, *p* < .001; SPLB: x^2^ (23) = 97.66, *p* < .001; FH: x^2^ (23) = 417.28, *p* < .001). Looking at Figure 8, there seems to be an increase in CIT across trials for both FH and SPLB but a decrease in CIT for the No eHMI condition. In the ‘yielding for the pedestrian’ condition, this increase in CIT indicates a more cautious behaviour of waiting until the deceleration became more prominent. Therefore, results suggest that pedestrians take a more cautious approach across trials with repeated exposure to FH in the ‘yielding for the pedestrian’ condition.

**Figure 9.**
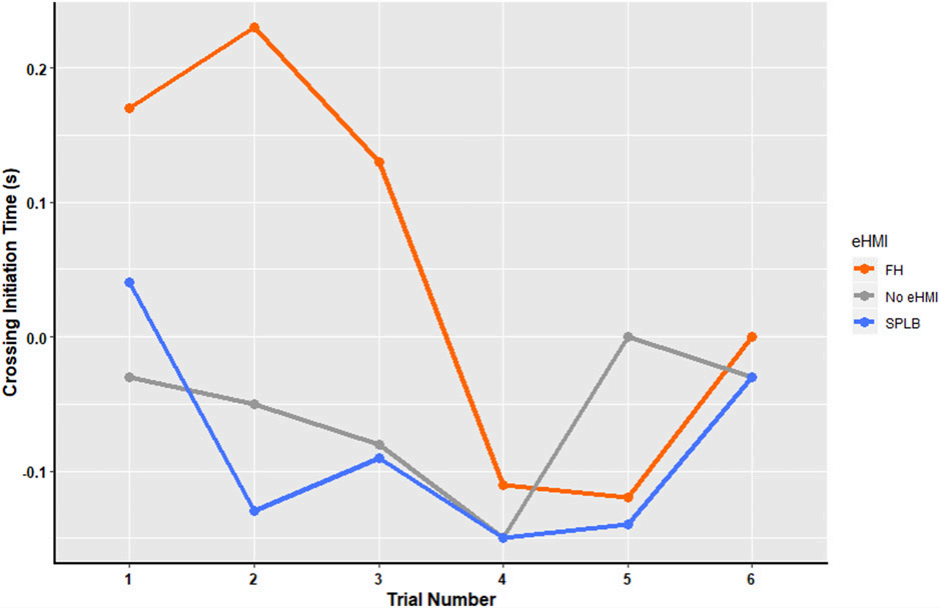
Mean of Crossing Initiation Time (s) across trials for FH, No eHMI, and SPLB for the ‘decelerating for the traffic lights’ condition.

For the ‘decelerating for the traffic lights’ condition (see Figure 9), there was a significant main effect of eHMI, x^2^ (2) = 8.29, *p* = .016. There was also a significant main effect of trial number x^2^ (5) = 11.89, *p* = .036, but no interaction was found. Pairwise comparisons showed significantly longer CIT in the FH (M = 0.18) condition, than the SPLB (M = 0.07) (*p* = .015), and No eHMI condition (M = 0.07) (*p* < .005); and a significant decrease in CIT across trials. It should be noted that a similar pattern of results emerges, even when the 5 participants who experienced collisions were excluded from the analysis, suggesting that this learning effect occurs for all participants. In this condition, the vehicle was not yielding to the pedestrian and, therefore, the safest behaviour was either to not cross at all, or to cross quickly once the gap emerged. Across all the conditions, approximately half of participants chose to cross (range = 52–66%). This number did not vary considerably across trials. However, the reduction in CIT across trials shows that increased experience led pedestrians to adopt safer crossing behaviours over time, crossing quickly or not at all.

## General Discussion, Conclusions, and Recommendations

In this study, we tested a use case where pedestrians were waiting to cross the road, and the approaching vehicle was either yielding for them, or decelerating for the traffic ahead. There were three main research questions in this paper. Our first research question was whether participants were able to identify the intention of the approaching vehicle. Results revealed more crossings were made when the vehicles were yielding for them, than when they were decelerating for the traffic ahead. This finding was in line with previous findings where pedestrians were able to make a judgement based on the vehicle’s implicit cues, such as its approaching speed, deceleration pattern and likely stopping position (i.e. Dey et al., 2021; Domeyer et al., 2020; Lee et al., 2021, 2022; Sheppard et al., 2023). A bimodal crossing pattern was observed, whereby pedestrians crossed when the approaching vehicles were either located very far away, or they waited until the vehicle came to a near stop, with little crossing occurring between these two distances (see also Giles et al., 2019; Lee et al., 2019; Lee et al., 2022; Markkula et al., 2020; Schneemann & Gohl., 2016). The only condition which resulted in some crossings between these two gaps was when the approaching AV was accompanied by a familiar eHMI (i.e. Flashing Headlights). However, this raised some questions about the dangers associated with such eHMIs, and whether pedestrians were interpreting the approaching vehicles’ implicit behaviour, or were blindly being influenced by the message conveyed by the familiar eHMI. This was addressed by our second research question: whether and how novel (i.e. Slow Pulsing Light Band) and familiar (i.e. Flashing Headlights) eHMIs affect pedestrians' ability to identify the intention of an approaching vehicle. Results showed that more crossings were made when the FH was presented, compared to the novel SPLB, and No eHMI, regardless of deceleration intention. Therefore, pedestrians were making crossing decisions based on the eHMI and were more influenced by the FH (in line with Lee et al., 2022, see also Kaleefathulah et al., 2020). Although FH was found to be visible earlier than SPLB, Lee et al. (2022) concluded that visibility did not appear to be the only reason for earlier crossings, with message familiarity and comprehension thought to play a role. FH is a commonly used signal to convey yielding intentions (John, 2004). This suggests that a more familiar eHMI, has a larger effect on pedestrians’ crossing decisions and behaviour, and can over-ride the message conveyed by the implicit behaviour of the AV. The power of the FH was seen for both yielding conditions, causing more collisions with the vehicles when these were actually decelerating for the traffic jam ahead. Clearly, this is an issue if such messages become the common method by which AVs communicate in the future. Kaleefathullah et al. (2020) found that explaining the meaning of a novel eHMI in advance, that is, increasing the clarity of the novel eHMI, had a similar effect on pedestrians behaviours, leading to over-trust in the eHMI (see Holländer, Colley, et al., 2019). Taken together these results suggest that pedestrians adopt a top-down information processing technique when making rapid decisions in traffic, interpreting the most salient piece of vehicle information (i.e. the eHMI) based on their previous knowledge and comprehension, leading to them sometimes ignoring relevant, but less salient, information from the environment, such as vehicle movement patterns. However, they do not infer such meanings from salient stimuli, without explicit instructions about meaning, or a large body of previous experience.

Some thought must be given to the timing and exact message provided by such eHMI, and how eHMI may influence pedestrians’ decision making. Future research, making use of subjective data to complement the findings of our objective measurements, may provide additional insights into why the FH signals had a larger impact on pedestrian behaviour than the SPLB, and whether a more cautious response to familiar eHMIs can be encouraged. The importance of designing efficient and optimal vehicle automation kinematics for communication should also be stressed (Domeyer et al., 2020; Sadigh et al., 2018).

Finally, the third research question investigated whether there were learning effects over time, in terms of how much pedestrians relied on the information conveyed through an eHMI. Results showed that pedestrians learned to take a more cautious approach across trials after realising that the presence of the FH did not always lead to a yielding vehicle. This observation is a positive outcome, as it shows that, after a few trials pedestrians were able to learn the message conveyed by the eHMI, but reverted to using the implicit behaviour of the AV for their crossing decisions. Similarly, a recent study showed that due to the higher exposure of unreliable turn indicators in Malaysia, Malaysian drivers are more attuned to implicit signals when judging intention of other drivers, whereas British drivers heavily rely on explicit signals (Sheppard et al., 2023). However, it is very important for AVs to provide accurate eHMIs to improve trust in AVs (Kaleefathullah et al., 2020), and further work needs to be conducted to ensure that there is no confusion between the implicit and explicit messages conveyed by future AVs. The scenario explored in this study is rather simple and artificial compared to the complexity of everyday crossing. Future studies should explore the impact of implicit and explicit communications in more complex scenarios, including multiple road users, variations in road users’ demographics and backgrounds, and different infrastructures (see also Madigan et al., 2023).

## Key Points

• We investigated how different deceleration intentions and how a novel or familiar eHMI affected pedestrians’ crossing behaviour.
• Pedestrians were more likely to cross when a familiar eHMI was presented, than the novel eHMI (or No eHMI) condition, regardless of the vehicle’s intention.
• A familiar eHMI could therefore lead to higher collision frequency, than the novel eHMI (or No eHMI), if the eHMIs were incongruent with the vehicles’ intention.
• Participants learned to take a more cautious approach across trials, and not to base their decisions solely on the familiar eHMI.
• It is important to provide eHMIs that are congruent with road users’ expectations, to achieve safe and acceptable interactions with AVs.
